# Single-Camera Three-Dimensional Digital Image Correlation with Enhanced Accuracy Based on Four-View Imaging

**DOI:** 10.3390/ma16072726

**Published:** 2023-03-29

**Authors:** Xinxing Shao, Jingye Qu, Wenwu Chen

**Affiliations:** Department of Engineering Mechanics, School of Civil Engineering, Southeast University, Nanjing 211189, China

**Keywords:** digital image correlation, multi-view geometric constraints, four-view imaging, pyramidal prism

## Abstract

Owing to the advantages of cost-effectiveness, compactness, and the avoidance of complicated camera synchronization, single-camera three-dimensional (3D) digital image correlation (DIC) techniques have gained increasing attention for deformation measurement of materials and structures. In the traditional single-camera 3D-DIC system, the left and right view images can be recorded by a single camera using diffraction grating, a bi-prism, or a set of planar mirrors. To further improve the measurement accuracy of single-camera 3D-DIC, this paper introduces a single-camera four-view imaging technique by installing a pyramidal prism in front of the camera. The 3D reconstruction of the measured points before and after deformation is realized with eight governing equations induced by four views, and the strong geometric constraints of four views can help to improve the measurement accuracy. A static experiment, a rigid body translation experiment, and a four-point bending experiment show that the proposed single-camera 3D-DIC method can achieve higher measurement accuracy than the dual-view single-camera 3D-DIC techniques and that the single-camera 3D-DIC method has advantages in reducing both random error and systematic error.

## 1. Introduction

Three-dimensional (3D) digital image correlation (DIC) is now a standard technique used to determine the mechanical properties of materials and structures [1,2]. In terms of the number of cameras used, the current 3D-DIC can be divided into traditional dual-camera 3D-DIC [1], single-camera 3D-DIC [3], and multi-camera 3D-DIC [4]. Owing to the advantages of cost-effectiveness, compactness, and the avoidance of complicated camera synchronization, single-camera 3D-DIC techniques have attracted increasing attention for deformation measurement. Pankow et al. developed a single-lens 3D-DIC system using a single camera and a series of mirrors and they applied the system for high-speed out-of-plane displacement measurements [5]. Genovese et al. presented a single-camera pseudo-stereo system using a bi-prism in front of the camera lens to split the scene into two equivalent lateral stereo views in the two halves of the sensor [6,7]. Xia et al. developed a diffraction-assisted image correlation for 3D displacement measurement using a single camera and 2D-DIC algorithm [8,9]. The color separation-based single-camera 3D-DIC using the 3CCD color camera or an industrial color CCD camera has also been developed [10,11]. In these single-camera 3D-DIC techniques, left and right-view images of the sample can be obtained directly with a single camera. After camera calibration, temporal matching, and stereo matching, the morphological information and 3D displacement of the specimens can be measured using a single camera. At present, the single-camera 3D-DIC techniques have been used in many applications, including vibration modal measurement [12], impact deformation measurement [13], video extensometer measurement [14], internal deformation measurement of pipelines [15], and single-event-camera based 3D trajectory measurement [16].

The single-camera 3D-DIC systems reported so far all use the two views for stereo imaging, namely the left view and the right view. Compared to the two-view imaging technique, the four-view imaging technique has been proven to improve the accuracy of 3D reconstruction [17,18]. In the traditional four-view imaging-based stereo vision system, four cameras are used to capture images from four different views [17]. The use of multiple cameras can give rise to problems such as high cost and complicated camera synchronization. Combining the single-camera 3D-DIC technique with the four-view imaging technique will be an effective way to improve the measurement accuracy without increasing the cost.

In this work, a single-camera four-view 3D-DIC technique is proposed for high-accuracy full-field deformation measurement to further enhance the measurement accuracy. By installing a pyramidal prism in front of the camera, the four different view images of the specimen can be easily obtained. The 3D reconstruction of the full-field object points before and after deformation is realized with eight governing equations induced by these four views, and the strong constraints of four views can help to improve the measurement accuracy. This paper focuses on improving the measurement accuracy of single-camera 3D-DIC for full-field displacement and strain measurement. Compared to the video extensometer [18], full-field deformation measurement has a very broad application prospect in the field of experimental mechanics.

The rest of the paper is organized as follows: The principles of the four-view imaging technique are described, and the governing equations for four-view 3D reconstruction are given in Section 2. In Section 3, to validate the effectiveness and accuracy of the proposed single-camera four-view 3D-DIC method, a static experiment, a rigid body translation experiment, and a four-point bending experiment was conducted, and the measurement results were analyzed and compared to the three-view and dual-view measurements. Conclusions are drawn in Section 4.

## 2. Methodology

**Figure 1:**
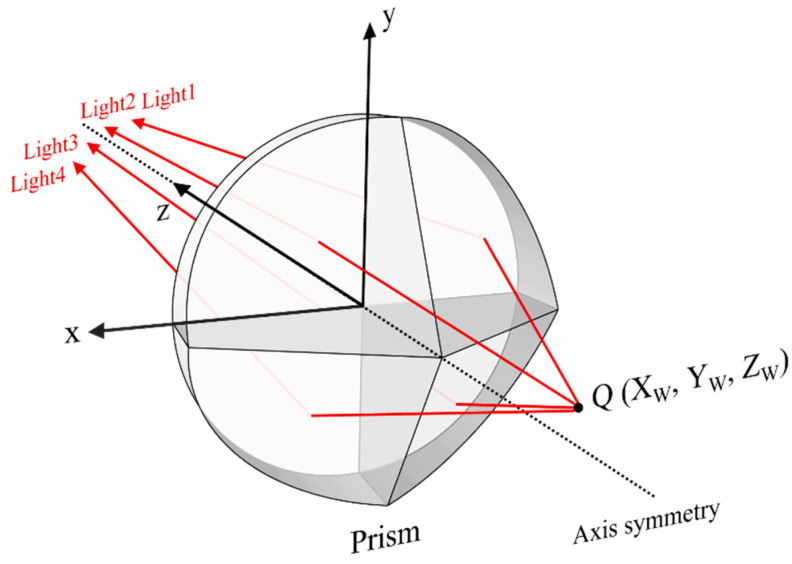
Basic schematic diagram of a pyramidal prism imaging path.

**Figure 2:**
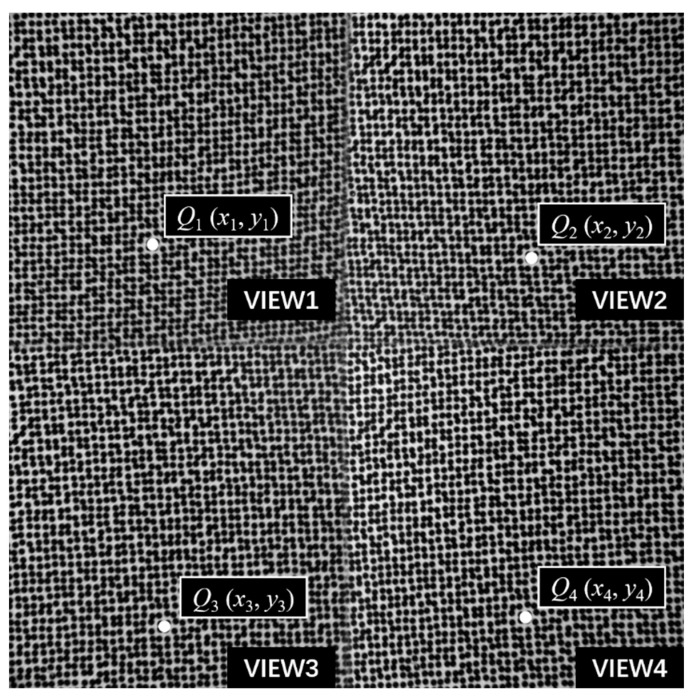
Speckle image of the specimen from four views.

Figure 1 shows the basic schematic diagram of a pyramidal prism imaging path. Through the refraction imaging of the pyramidal prism, the image of four views of the specimen can be obtained. If a camera is placed behind the pyramidal prism, the speckle image of the specimen from four views can be recorded for the single-camera 3D-DIC measurement, as shown in Figure 2. In detail, the initial field of view is divided into four equally sized sub-views. Ideally, the measured object center, the prism center, and the optical center of the camera are aligned. It is strictly 50% vertical and 50% horizontal without considering device layout errors.

**Figure 3:**
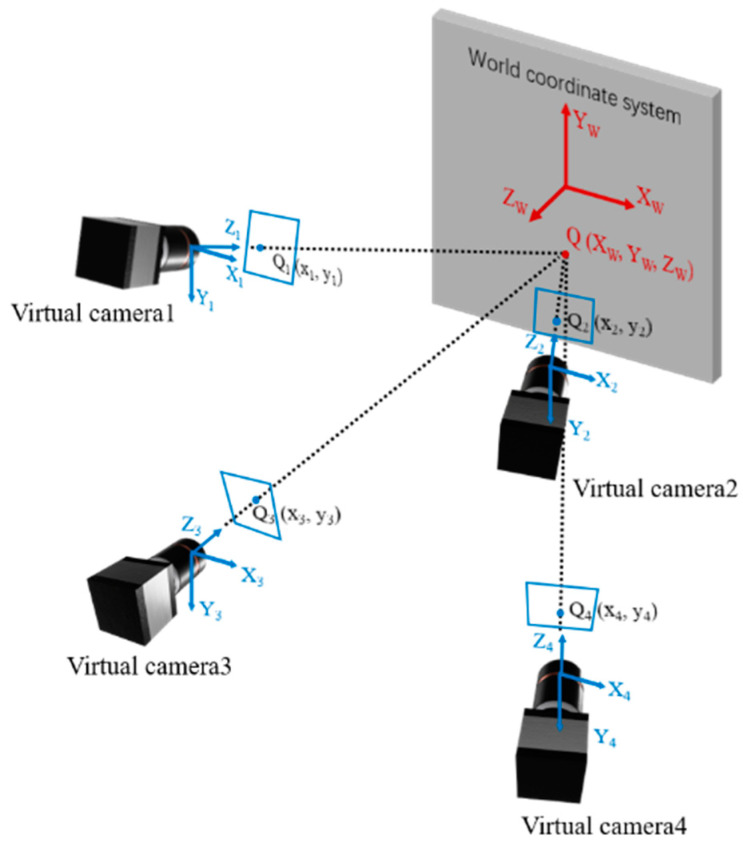
Schematic diagram of the four-view stereo vision.

Figure 3 shows the schematic diagram of the four-view stereo vision, the four virtual cameras represent shots from different views. The point Q is the object point to be measured and points $Q_{1}$, $Q_{2}$, $Q_{3}$ and $Q_{4}$ are the image points projected onto the image from four different views. With lens distortion removal through the distortion parameters obtained from the four-view calibration, the projection equation can be written as follows,

(1) $$\left\{\begin{array}{l} x^{i}=f_{x}\frac{r_{11}^{i}X_{W}+r_{12}^{i}Y_{W}+r_{13}^{i}Z_{W}+t_{x}^{i}}{r_{31}^{i}X_{W}+r_{32}^{i}Y_{W}+r_{33}^{i}Z_{W}+t_{z}^{i}}+f_{s}^{i}\frac{r_{21}^{i}X_{W}+r_{22}^{i}Y_{W}+r_{23}^{i}Z_{W}+t_{y}^{i}}{r_{31}^{i}X_{W}+r_{32}^{i}Y_{W}+r_{33}^{i}Z_{W}+t_{z}^{i}}+c_{x}^{i}\\ y^{i}=f_{y}\frac{r_{21}^{i}X_{W}+r_{22}^{i}Y_{W}+r_{23}^{i}Z_{W}+t_{y}^{i}}{r_{31}^{i}X_{W}+r_{32}^{i}Y_{W}+r_{33}^{i}Z_{W}+t_{z}^{i}}+c_{y}^{i} \end{array}\right.,i=1,2,3,4;$$

where $\left(\begin{array}{ccc} X_{W} & Y_{W} & Z_{W} \end{array}\right)$ are the world coordinates of point Q, $\left(x^{i},y^{i}\right),i=1,2,3,4$ are the image coordinates of points $Q_{1}$, $Q_{2}$, $Q_{3}$ and $Q_{4}$, $\left(c_{x}^{i},c_{y}^{i},f_{x}^{i},f_{y}^{i},f_{s}^{i}\right)$ are the intrinsic parameters of the single camera, $\left(c_{x}^{i},c_{y}^{i}\right)$ is the principle point coordinate which is the intersection point of the camera optical axis and the sensor, $f_{x}^{i},f_{y}^{i}$ is equivalent focal length, and $f_{s}^{i}$ represents the skew of the camera sensor. ${\left[t_{x}^{i},t_{y}^{i},t_{z}^{i}\right]}^{T}$ is the translation vector and $r_{11}^{i}$ to $r_{33}^{i}$ are the rotation matrix parameters which can be transformed from a rotation vector by using Rodrigues’ transformation formula, which represents the rotation matrix from the upper left view coordinate system to the i-th view coordinate system, and the superscripts i denote the i-th view.

According to Equation (1), the 3D coordinates of the object point can be solved by the least square method, and the solving equation is as follows:

(2) $$\begin{array}{c} \left[\begin{array}{ccc} x^{i}r_{31}^{i}-c_{x}^{i}r_{31}^{i}-f_{s}^{i}r_{21}^{i}-f_{x}^{i}r_{11}^{i} & x^{i}r_{32}^{i}-c_{x}^{i}r_{32}^{i}-f_{s}^{i}r_{22}^{i}-f_{x}^{i}r_{12}^{i} & x^{i}r_{33}^{i}-c_{x}^{i}r_{33}^{i}-f_{s}^{i}r_{23}^{i}-f_{x}^{i}r_{13}^{i}\\ y^{i}r_{31}^{i}-c_{y}^{i}r_{31}^{i}-f_{y}r_{21}^{i} & y^{i}r_{32}^{i}-c_{y}^{i}r_{32}^{i}-f_{y}^{i}r_{22}^{i} & y^{i}r_{33}^{i}-c_{y}^{i}r_{33}^{i}-f_{y}^{i}r_{23}^{i} \end{array}\right]\left[\begin{array}{c} X_{W}\\ Y_{W}\\ Z_{W} \end{array}\right]\\ =\left[\begin{array}{c} f_{x}^{i}t_{x}^{i}+f_{s}^{i}t_{y}^{i}+c_{x}^{i}t_{z}^{i}-x^{i}t_{z}^{i}\\ f_{y}^{i}t_{y}^{i}+c_{y}^{i}t_{z}^{i}-y^{i}t_{z}^{i} \end{array}\right],i=1,2,3,4 \end{array}$$

It can be seen from Equation (2) that a total of eight equations can be used to solve the three unknowns of 3D coordinates of the object point. The 3D coordinates of a point will be determined by the intersection of four rays. Compared to the traditional dual-view single-camera 3D-DIC, there are two more ray geometric constraints. The four equations brought by the two-ray geometric constraints can reduce the uncertainty of 3D reconstruction [14].

**Figure 4:**
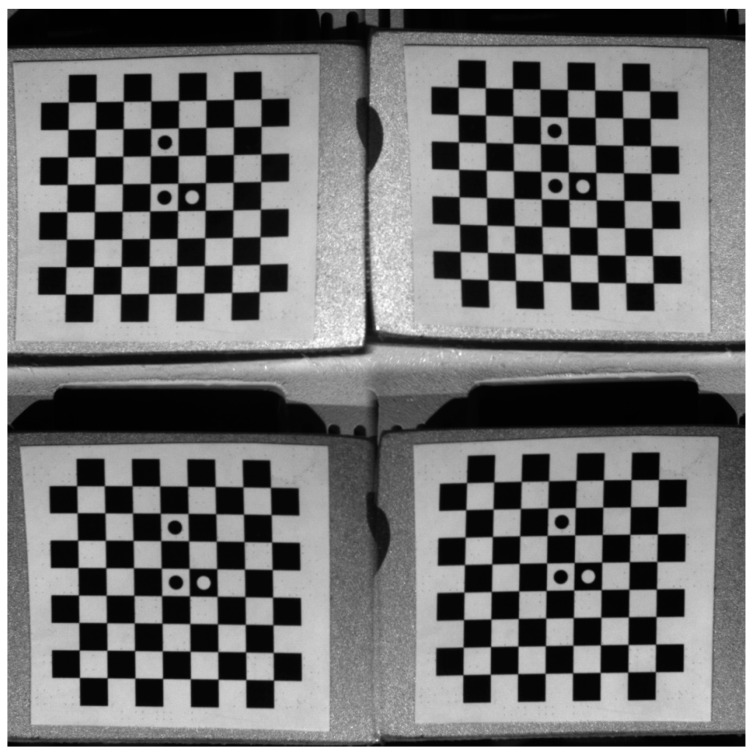
Calibration image of a chessboard calibrator from four views.

By calibrating the four-view single-camera 3D-DIC, the intrinsic parameters, and the extrinsic parameters between the second, third, and fourth views with the first view can be obtained. A set of photos of a planar checkerboard calibration board with different poses were captured by four virtual cameras at the same time, then the intrinsic parameters and the extrinsic parameters of cameras were calculated using Zhang’s calibration method [19]. An example of calibration images is shown in Figure 4.

**Figure 5:**
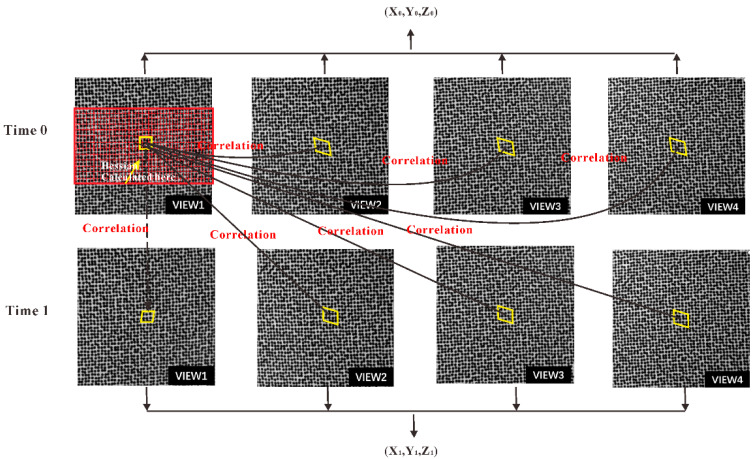
Four-view stereo vision matching and sequence image matching.

Figure 5 demonstrates the stereo image matching among the four cameras and sequence image matching for the same camera. When both image matching and calibration are completed, the morphologic information of the objects can be obtained, as shown in Figure 3. Then, the 3D displacements can be calculated with the 3D coordinates of the same point before and after deformation. Based on the reconstructed 3D shape and the calculated 3D displacement, the local surface strains can be computed. Once the initial topology of the specimen has been measured, the normal direction of each point in the initial topology can be defined. Using a least-square plane to fit the data array around each point, the local surface of each point can be defined. According to the local surface and X direction of the Lagrange world coordinate system after the 3D reconstruction, the local coordinate system can be defined. Then, all three components of displacements for each point on the surface are projected to the local Cartesian coordinate system and the strain can be calculated using least-square method.

## 3. Experimental Results

To validate the effectiveness and accuracy of the proposed single-camera four-view 3D-DIC, a static experiment, a rigid body translation experiment, and a four-point bending experiment were conducted, respectively. In the static experiment and the translation experiment, the displacement accuracy in both in-plane and out-of-plane directions was verified. In the four-point bending experiment, the strain field was measured and compared to the strain gauge technique.

**Figure 6:**
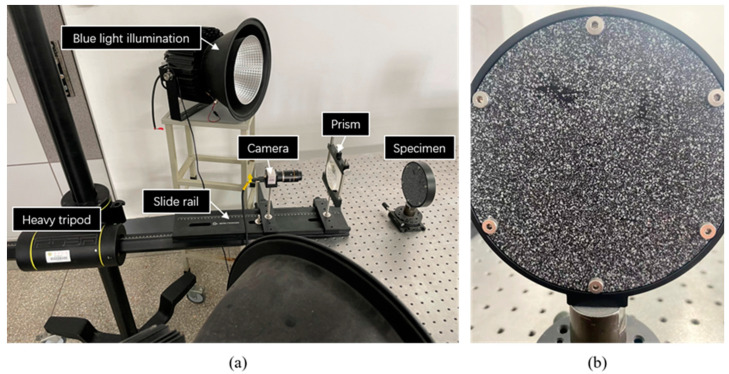
Static experiment for: (**a**) experiment setup; (**b**) specimen.

**Figure 7:**
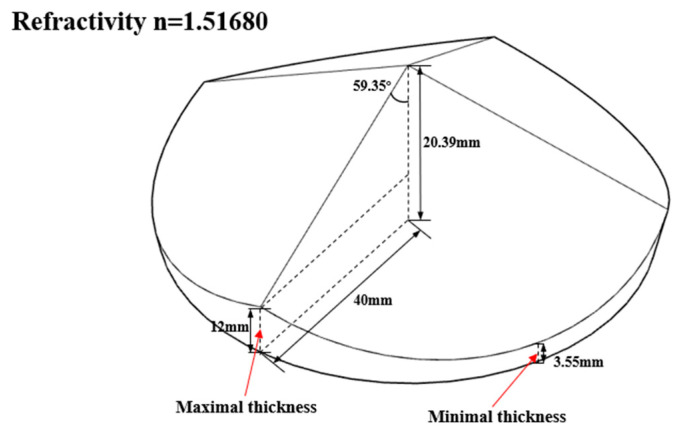
The geometry of the pyramidal prism.

As shown in Figure 6, a single-camera four-view 3D-DIC system consisting of a single camera and a pyramidal prism was mounted on the slide rail (Daheng Optics, GCM-72) on a heavy tripod (FOBA, ASLAI). It is worth mentioning that poor mechanical stability of the setup can affect the final camera calibration results in the same way as lens shakes and the motion blur of calibration images [20,21]. The heavy tripod and the slide rail had to have excellent stability to ensure the accuracy of experiments. The geometry of the pyramidal prism is shown in Figure 7 and the distance between the pyramidal prism and lens was 105 mm. The detailed system configuration is listed in Table 1 and the extrinsic parameters between the first view and other views are shown in Table 2. To reduce the effect of ambient light and refraction, the blue light source and the narrow band filter were used.

As shown in Figure 7, the prism’s angle designed is 59.35°. In the calculation of the geometric sizes of the prism, the field of view size, the prism diameter, and the distance between the object and the prism are predetermined, then the angle can be calculated. Actually, the presented prism with the given angle is not the only possible configuration. The smaller the prism’s angle, the greater the ability of the prism to converge light and therefore the larger the field of view. When all other dimensions remain the same, a larger field of view can be obtained by reducing the prism angle appropriately. We recommend that the angle be preferably less than the designed 59.35°, otherwise the field of view will be too small. However, this angle cannot be reduced below about 40° as the total reflection will occur in the prism.

**Figure 8:**
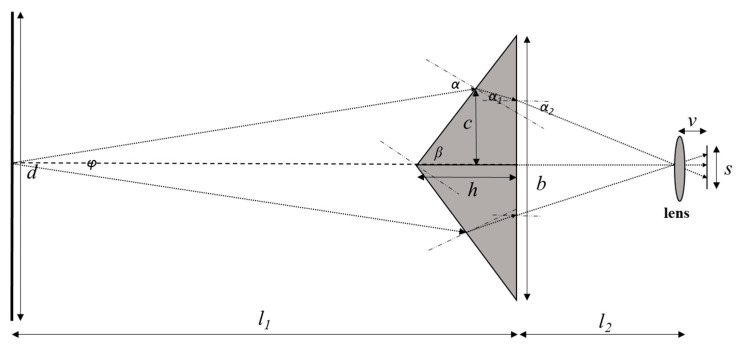
The geometric optical path diagram of the pyramidal prism.

Figure 8 shows the geometric optical path diagram of the pyramidal prism. The relation between the geometric parameters can be calculated according to Equations (3)–(6),

(3) $$\tan\alpha_{2}=\frac{s}{4v}$$

(4) $$\frac{\sin\alpha_{2}}{\sin\alpha_{1}}=n$$

(5) $$\frac{\sin\alpha }{\sin\left(90-\beta -\alpha_{1}\right)}=n$$

(6) φ=2(α−(90−β))

where *s* is the size of the imaging chip, *v* is the distance between the lens and the camera sensor, *n* is the refractivity, φ is the rotation angle, $\alpha ,\alpha_{1},\alpha_{2}$ are the angles of refraction. In the current manuscript, the *s* = 11.264 mm, *v* ≈ 20 mm, *n* = 1.5168, β = 59.35, so the rotation angle φ is close to 16°, which is consistent with the calibrated value in Table 2.

Further, the distance between the prism and the lens set and the distance between the prism and the object set can also be roughly calculated according to Equations (7) and (8),

(7) $$\frac{s}{2v}\approx \frac{d}{\left(l_{1}+l_{2}\right)}$$

(8) $$\frac{l_{1}}{l_{2}}\approx \frac{2\sin\beta d}{b}$$

where d is the field of view, $l_{1}$ is the distance between the prism and the object set, $l_{2}$ is the distance between the prism and the lens set. In the current manuscript, the d = 100 mm, so the $l_{1}+l_{2}$ is about 300 mm and $l_{1}$ is about 200 mm, and $l_{2}$ is about 100 mm.

**Figure 9:**
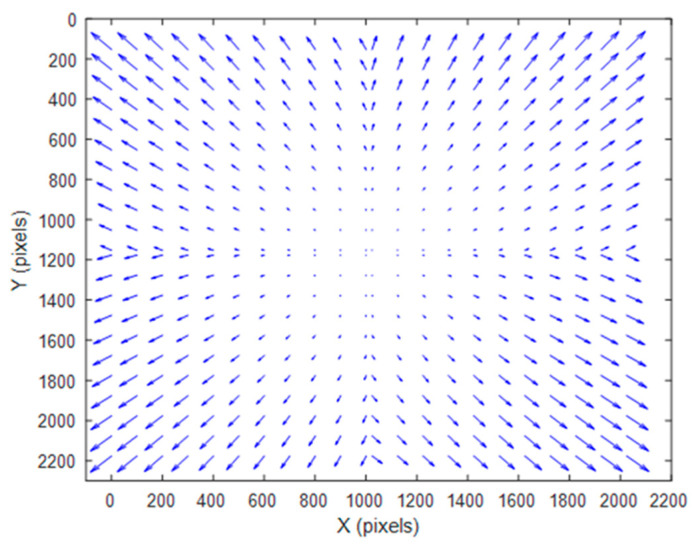
Distortion calibration plot of the single-camera four-view 3D-DIC system.

A conventional five-parameter distortion model is used in this work. It consists of three radial and two tangential distortion parameters. The prism and the camera lens are treated as a lens set. We assume that the conventional distortion model can correct the overall distortion. The distortion calibration plot of the single-camera four-view 3D-DIC system is shown in Figure 9.

**Figure 10:**
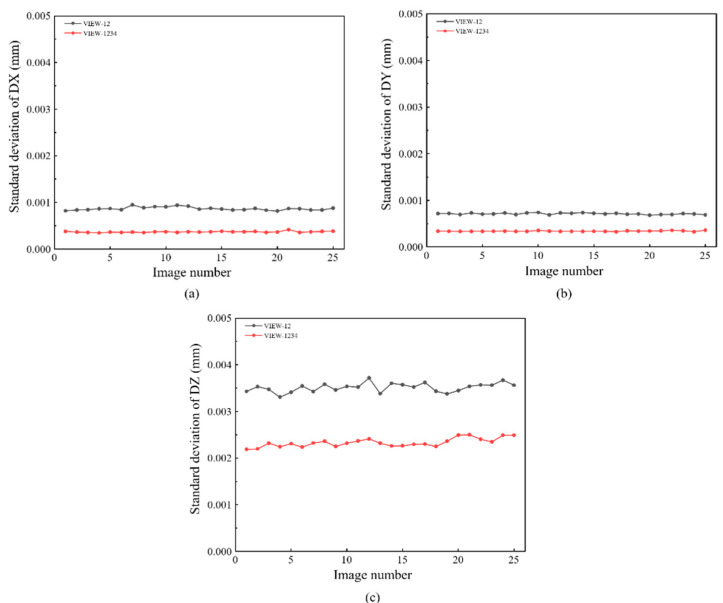
Comparisons of displacement standard deviation in static experiment between case VIEW-12 (results obtained from camera1 and camera2) and case VIEW-1234 (results obtained from four cameras): (**a**) DX; (**b**) DY; (**c**) DZ.

The static experiment configuration is shown in Figure 6a. Artificial speckles are generated on the surface of the specimen in Figure 6b. The working distance between the specimen and the prism is 200 mm. Twenty-five images are captured by the single four-view camera system and calculated by DIC. In detail, VIEW 1 and VIEW 2 are used to construct a conventional two-view DIC, and all four views are combined to provide a four-view DIC measurement. The standard deviation error of displacement for different directions (DX, DY, and DZ) are shown in Figure 10, respectively. Practically, the random error is represented by the standard deviation error. It is noticed that four-view DIC shows a distinct improvement in the random error. Compared to the two-view measurement, the in-plane displacement random error of the four-view measurement is reduced by 0.0004 mm. For out-of-plane displacement random error, it is reduced from 0.0035 mm to 0.002 mm. It can be noticed that the random error is obviously reduced by the strong geometric constraints of four views.

**Figure 11:**
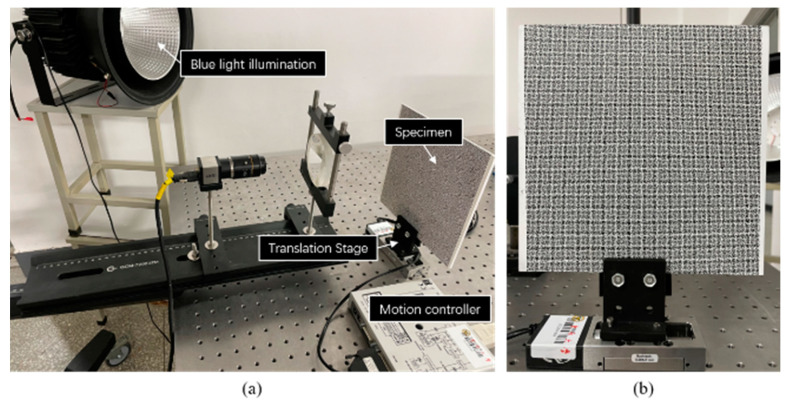
Translation experiment for: (**a**) experiment setup; (**b**) specimen and translation stage.

The translation experiment configuration is shown in Figure 11a. A translation stage (Newport, MFA-CC) controlled by a motion controller (Newport, SMC100) drove the specimen to move in a set direction. The specimen and the translation stage are shown in Figure 11b. Speckle patterns on the specimen were generated by applying the water transfer printing (WTF) technique [22], and the diameter of each speckle was 1 mm. In the experiment, the translation stage moved 0.5 mm for each step with a total of eight steps. Under each translation, twenty images were captured averagely to reduce the influence of environmental disturbances. In this experiment, the dual-view, three-view, and four-view single-camera 3D-DIC were used to calculate the displacement field.

**Figure 12:**
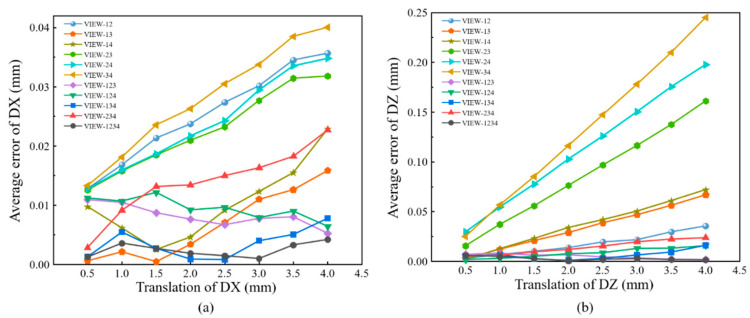
Results and comparisons in the rigid body translation experiment: (**a**) absolute error of in-plane displacement; (**b**) absolute error of out-of-plane displacement.

For comparisons, full-field displacements of each translation are averaged to calculate the absolute bias error, as shown in Figure 12. It can be seen in Figure 12a that the error of in-plane displacement based on four-view 3D-DIC is the smallest. For four-view 3D-DIC, the average error of all translations is about 0.002 mm, and the error keeps relatively stable. While the results of three-view 3D-DIC show a lower accuracy, the average error is about 0.01 mm. For the dual-view 3D-DIC, the VIEW14 has the smallest systematic error and the absolute bias error can reach 0.02 mm. For out-of-plane displacement, four-view 3D-DIC also achieves the highest accuracy and the average error is about 0.003 mm. For the three-view and dual-view 3D-DIC, the error rises to 0.009 mm and 0.07 mm, respectively. The translation experiment effectively proves the high accuracy and robustness of the single-camera four-view 3D-DIC. Actually, compared to the two-view system, almost an order of magnitude reduction in displacement error in the four-view system is mainly due to inaccurate calibrations. It can be seen in Figure 12 that there is a noticeable increase in error with the increasing translation of DX and DZ in two- and three-view systems. However, the error in the four-view system remains relatively stable. The impact of calibration on the different view systems will be further analyzed in our future work.

**Figure 13:**
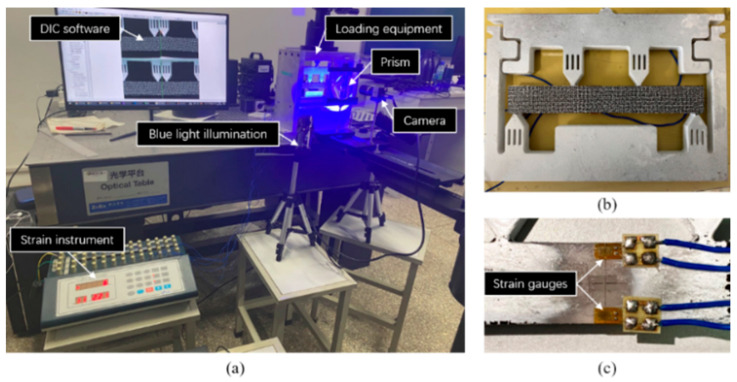
Four-point bending experiment: (**a**) experiment setup; (**b**) specimen; (**c**) strain gauge.

**Figure 14:**
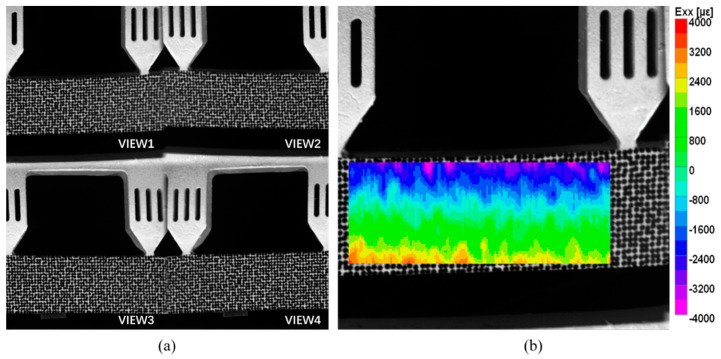
(**a**) Speckle image of the four-point bending beam from four views in the experiment; (**b**) full-field strain $\varepsilon_{xx}$ of the pure bending section.

Figure 13a shows the setup of the four-point bending experiment, and the specimen is shown in Figure 13b. The four-point bending beam was 150 mm long and 20 mm wide. Speckle patterns were also made via the WTP technique, and the speckle diameter was 0.8 mm. As shown in Figure 13c, the strain gauges were attached to the middle edges of the beam to provide the reference truth of strain. The world coordinate system after 3D reconstruction was transformed to the front surface of the specimen, and the X and Y directions coincide with the horizontal and vertical directions of the beam, respectively. Therefore, since the size of the gauges was known, the frontal region corresponding to the strain gauge grid area could be selected precisely for comparison. The specimen was loaded under ten different load levels. Under each load, twenty images were captured averagely to reduce the influence of environmental disturbances. The four-view speckle image is shown in Figure 14a. Then, the dual-view, three-view, and four-view single-camera 3D-DIC were used to calculate the strain field. The strain field in the pure bending section by the four-view 3D-DIC is presented in Figure 14b. The direction of the strain component Exx is the horizontal direction of the specimen. Due to the presence of subsets in the displacement and strain calculations, half of the subset size close to the upper and lower boundary of the specimen cannot be calculated. It should be noted that, although the strain gauges are placed at the specimen edge, there was still some distance between the grid and the specimen edge. The strain achieved by the strain instrument was actually the average strain in the central grid of the strain gauge. As a result, the strain of the frontal region corresponding to the strain gauge grid could be fully measured.

**Figure 15:**
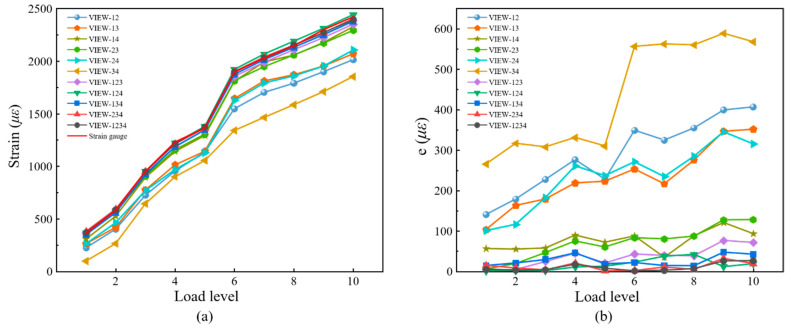
Results and comparisons in the four-point bending experiment: (**a**) measured strain; (**b**) absolute error.

For comparison, the average strain of the speckle region corresponding to the strain gauge was extracted. The strain attained by 3D-DIC and the strain gauge is shown in Figure 15a, and the absolute errors between 3D-DIC and strain gauge are shown in Figure 15b. It is worth mentioning that only the strain gauge at the lower edge, i.e., at the tensile end, is analyzed in Figure 15. It can be seen in Figure 15 that the four-view 3D-DIC always achieves the highest accuracy of strain (error below 20 με) and the error keeps relatively stable under different loads. This solidly reveals the precision and stability of the single-camera four-view 3D-DIC system. In Figure 15b, the strain error of three-view 3D-DIC is about 50 με, while the dual-view 3D-DIC lead to a bigger error. In the dual-view 3D-DIC, VIEW14 has the smallest systematic error and the absolute error can even achieve 100 με. The four-point bending experiment effectively proves the high accuracy and robustness of the single-camera four-view 3D-DIC.

## 4. Discussion and Conclusions

**Figure 16:**
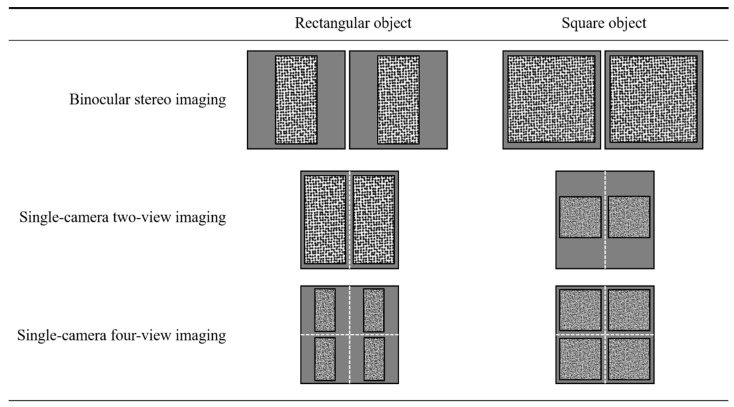
Resolution comparison of binocular stereo imaging, single-camera two-view, and four-view imaging for rectangular and square objects.

Compared to traditional binocular stereo systems, the pseudo stereo image captured by single-camera 3D-DIC techniques typically suffers considerable resolution reduction. To demonstrate this clearly, a resolution comparison of binocular stereo imaging, traditional single-camera 3D-DIC based on two-view imaging, and the proposed accuracy-enhanced single-camera 3D-DIC based on four-view imaging is shown in Figure 16. When the measured objects are rectangular, the traditional two-view imaging shows the same resolution compared with the binocular system, while the four-view system suffers resolution loss. In this case, the four-view system improves the measurement accuracy, but the reduction in resolution reduces it, which is a contradiction. When the object comes to a square, the single-camera two-view and four-view imaging systems both show reduced resolution. However, there is no resolution difference between the proposed four-view system and the conventional two-view system when measuring square objects. Actually, we aim to compare the proposed method with the two-view single-camera 3D-DIC techniques, not the traditional binocular stereo systems. It can be noticed that the proposed four-view system enjoys the advantages of higher accuracy and no resolution loss when measuring square objects. Resolution reduction is one of the inherent drawbacks of single-camera 3D-DIC techniques, and the central region with the best image quality of lens and camera sensor is underutilized.

Despite these drawbacks, single-camera 3D-DIC techniques have gained increasing attention for deformation measurement due to their outstanding advantages of cost-effectiveness, compactness, and avoidance of complicated camera synchronization. Actually, in terms of accuracy, precision, and error reduction, the setup proposed does not have obvious advantages over the previously reported four-camera setup [17]. However, compared to the previously four-camera system, the method in this work still has some significant advantages:

(1) Due to the compactness of the setup proposed in this work, the rigid connection between devices is easier to achieve than the four-camera system, and it is very important for measurement accuracy;

(2) Hard synchronization of four cameras requires additional synchronous trigger instruments, which greatly increases the complexity and cost of the system;

(3) In some cases where the experimental space is limited, the method in this work has the advantage of being more flexible, such as the experiment of observing experimental objects through a small window in a high-temperature box.

In summary, an accuracy-enhanced single-camera 3D-DIC technique is proposed based on four-view imaging. By installing a pyramidal prism in front of the camera, the speckle image of four different views of the specimen can be obtained. The basic principles and system configuration are described in detail. Both the rigid body translation experiment and four-point bending experiment show that the proposed single-camera 3D-DIC method can achieve higher measurement accuracy than the dual-view single-camera 3D-DIC and the three-view single-camera 3D-DIC techniques. In the four-point bending experiment, the absolute strain errors of the proposed four-view single-camera 3D-DIC are less than 20 με. In general, the setup is an interesting generalization of a camera being coupled with Risley prisms scanners [23,24,25]. Although the proposed single-camera 3D-DIC method has some limitations, such as the decreased field of view and increased computation cost, the application of the single-camera 3D-DIC method will still be greatly expanded with the improvement of their accuracy.

## Figures and Tables

**Table 1:** System parameters for the experiments.

Camera	IDS, Ueye CP3370
Resolution/Pixel size	2048 × 2048 pixels/5.5 μm
Lens	Kowa, 25 mm
Working distance	200 mm
Field of view	100mm
Subset/Step	27 × 27 pixels/5 pixels
Illumination/Wavelength	Single narrowband blue light/450 nm

**Table 2:** Extrinsic parameters between the first view with other views.

External Parameters	View 1–2	View 1–3	View 1–4
Rotation-X	0.14°	15.77°	15.74°
Rotation-Y	−15.53°	0.65°	−15.89°
Rotation-Z	3.61°	0.99°	0.17°
Translation-X	−55.27 mm	2.85 mm	−57.03 mm
Translation-Y	−3.21 mm	−57.15 mm	−57.15 mm
Translation-Z	−6.98 mm	−1.74 mm	−6.12 mm

## Data Availability

The data that support the findings of this study are available from the corresponding author upon reasonable request.
